# Integrated sequencing of exome and mRNA of large-sized single cells

**DOI:** 10.1038/s41598-017-18730-y

**Published:** 2018-01-10

**Authors:** Lily Yan Wang, Jiajie Guo, Wei Cao, Meng Zhang, Jiankui He, Zhoufang Li

**Affiliations:** Department of Biology, Southern University of Science and Technology, Shenzhen, 518055 China

## Abstract

Current approaches of single cell DNA-RNA integrated sequencing are difficult to call SNPs, because a large amount of DNA and RNA is lost during DNA-RNA separation. Here, we performed simultaneous single-cell exome and transcriptome sequencing on individual mouse oocytes. Using microinjection, we kept the nuclei intact to avoid DNA loss, while retaining the cytoplasm inside the cell membrane, to maximize the amount of DNA and RNA captured from the single cell. We then conducted exome-sequencing on the isolated nuclei and mRNA-sequencing on the enucleated cytoplasm. For single oocytes, exome-seq can cover up to 92% of exome region with an average sequencing depth of 10+, while mRNA-sequencing reveals more than 10,000 expressed genes in enucleated cytoplasm, with similar performance for intact oocytes. This approach provides unprecedented opportunities to study DNA-RNA regulation, such as RNA editing at single nucleotide level in oocytes. In future, this method can also be applied to other large cells, including neurons, large dendritic cells and large tumour cells for integrated exome and transcriptome sequencing.

## Introduction

Integrated single-cell exome and transcriptome data can address many questions, including somatic variation, meiotic recombination, cell-to-cell heterogeneity in gene expression and DNA-RNA regulation. Despite the rapid technique advances for single-cell sequencing, only a few studies have addressed both the genome and transcriptome of a single cell^1–3^. These studies presented significant technical advances in simultaneous single-cell genome/transcriptome profiling. They successfully separated RNA from genomic DNA (gDNA) of the same cell, either by capturing mRNA with magnetic beads and collecting gDNA from lysed supernatants^1^, or by releasing the cytoplasm from the cell while keeping the nucleus completely intact^2^. Then they used comparative genomic hybridization and cDNA array analysis^1^, or targeted sequencing of selected genes and transcripts^2^, to reveal the connections between the genotype and phenotype of a single cell. The focus of these studies was on improving the separation of RNA and DNA, especially the work by Shintaku *et al*.^3^, who used electric fields to separate DNA and RNA and then quantified the separated molecules. However, the profiling of the genome and transcriptome was limited to a few genes and at a low resolution. For instance, G&T-seq and DR-seq are only able to investigate the copy number variation of single cells due to loss of DNA and RNA during separation step, or the low efficiency of binding affinity^4,5^. Techniques of revealing single-nucleotide resolution of DNA-RNA regulation in single cells are highly demanding and with broad interests, such as SNP calling in single cells or capturing RNA editing events at single-cell level.

RNA editing is a process that specific nucleotides in RNA sequences are changed after transcription. RNA editing in mRNA alters the amino acid sequence of the encoded protein so that it differs from the protein predicted by the genomic DNA sequence. The editing events can be divided into two categories, one called insertion/deletion editing, and the other called substitution (usually A to I and C to U) of nucleotides within the RNA molecule caused by an adenosine deaminase (ADAR) enzyme^6^. The editing process is related to RNA degradation or evolution^7,8^. However, the function of this process is far from understood. There is no report on RNA editing based on single-cell sequencing data so far.

We propose a new method that can be applied to large-sized cells, including oocytes (~100 μm), large neurons (50–100 μm, such as motor neuron), hair cells (~50 μm), dendritic cells (20–50 μm) and large tumour cells (~30 μm). Separating DNA and RNA of a single cell by microinjection is a well-practised technique, and it is widely used in oocytes and neurons^9–12^. Microinjection can keep the nucleus intact, with no DNA loss, to maximize the amount of raw DNA and RNA material. Following microinjection, we conducted single cell genome amplification followed by exome-seq on the isolated nucleus and single cell mRNA amplification followed by mRNA-seq on the enucleated cytoplasm to achieve the goal of integrated DNA-RNA sequencing. For a pilot study, we performed exome-seq and mRNA-seq on six secondary oocytes from one mouse. As a comparison, we also sequenced their counterpart polar bodies (PBs), three intact oocytes, and bulk liver cells from the same mouse, as well as a 200-mixed-oocytes population from several mice. We detected a similar number of expressed genes in enucleated single oocytes and intact single oocytes, indicating that our method does not lead to a significant loss of mRNA transcripts. The expression values of single enucleated oocytes correlated highly with those of intact oocytes, but showed low correlation with that of bulk oocytes, suggesting the heterogeneity of individual cells. By integrating the exome and transcriptome profiles in a single cell, we obtained informative results on RNA editing, which shed light on the connection between genotype and phenotype of a single cell.

## Methods

### Ethics statement

This study was approved by Southern University of Science and technology (SUSTC). All the experiments were performed in accordance with guidelines and regulations of the SUSTC. All methods are approved by the committee of SUSTC and carried out in accordance with relevant guidelines and regulations of SUSTC. All the analysis was performed anonymously.

### Sample collection and preparation

Meiosis II (MII) oocytes were collected from superovulated sexually mature female Kunming mice. The mouse oviducts were dissected and placed in M2 media (CytoSpring, CA, USA), and the cumulus cell complex extracted. Cumulus cells around the oocytes were then removed by treatment with hyaluronidase (Sigma, MO, USA), and the oocytes washed by pipetting them 4–6 times with M2 media. Oocytes were then collected under a stereomicroscope by mouth pipetting. Thereafter, oocytes were manipulated using a microinjection system (Eppendorf, NY, USA). Next we transferred the oocytes to a drop of M2 media under mineral oil (Sigma, MO, USA) in a 3.5-cm dish and partially removed the zona pellucida by laser-assisted biopsy. We collected the first polar body and nucleus in a micropipette into a 0.2 mL PCR tube with 5 μL nuclease-free distilled water. We then collected the cytoplasm (oocyte without nucleus or first polar body (PB1) into a 0.2 mL PCR tube with 5 μL lysis buffer. The nucleus, first polar body and cytoplasm were stored at −80 °C until required for library preparation. Negative controls were either nuclease-free water or lysis buffer alone.

### Single cell DNA amplification

We amplified genomic DNA from single isolated nuclei. Out of six different samples, two samples (S1 and S2) were amplified using the REPLI-g Single Cell Kit (Qiagen, Hilden, Germany) based on the multiple displacement amplification (MDA) method. In short, a single nucleus was lysed and denatured at 65 °C for 10 minutes. DNA amplification was then performed with random hexamer primers binding to the template and incubation at 30 °C for 8 hours with the high fidelity ϕ29 DNA polymerase. The reactions were then inactivated at 65 °C for 3 minutes and stored at −80 °C. The other four samples (S3, S4, S5 and S6) were amplified using the GenomePlex single cell amplification kit WGA4 (Sigma, MO, USA) based on degenerate-oligonucleotide-primed PCR (DOP-PCR). In short, we lysed the nucleus and digested proteins with protease K at 50 °C for 1 hour. The genomic DNA was then fragmented into 200–400 bp fragments at 99 °C for 4 minutes. Random primers linked with common adaptors were annealed to the fragmented DNA template using the following incubation: 16 °C for 20 minutes, 24 °C for 20 minutes, 37 °C for 20 minutes, 75 °C for 5 minutes, and stored at 4 °C. Then, amplification was performed with an initial denaturation at 95 °C for 3 minutes, and 25 cycles of 94 °C for 30 seconds and 65 °C for 5 minutes. The amplified products were purified using Qiagen PCR purification reagents (Qiagen, Hilden, Germany). The sequencing libraries were constructed by BGI-Shenzhen and sequenced using Illumina HiSeq 2000 sequencing platform.

### Exome-seq

We used SureSelect^QXT^ exome enrichment kit (Agilent technologies, CA, USA) to capture and enrich exome regions from the sequencing library of single-cell genomic DNA. In short, we mixed the sequencing library from a single nucleus with SureSelect exome probes, which were tagged with magnetic labels, and incubated at 65 °C for 24 hours or longer (optimal, 72 hours), allowing the probe to thoroughly hybridized to the library. Then the purified sequences were further amplified by PCR and purified. After quantity assessment using a bioanalyzer, fragments were sequenced on Illumina HiSeq 2000 sequencing platform (Illumina, CA, USA).

### Single cell RNA amplification

The single cell transcriptome of enucleated cytoplasm was amplified using SMARTer ultra low RNA kit (Clontech, CA, USA) according to the manufacturer’s instructions. Briefly, we first synthesized the first strand cDNA from single enucleated cytoplasm using modified oligo (dT) (SMART CDS primer) and then tailed several additional nucleotides to 3′ end of the first strand cDNA using the enzyme’s terminal transferase activity. A common adaptor (SMARTer II A oligonucleotide) was linked to 3′ end of the first strand cDNA. The resulting full-length single strand cDNAs started with poly(T) and ended with a common adaptor. The entire transcriptome of the enucleated cytoplasm was then amplified using universal primers (common adaptor and oligod(T)) for sequencing library construction. The sequencing libraries were constructed by BGI-Shenzhen and sequenced using Illumina HiSeq 2000 sequencing platform.

### Analysis of DNA sequence data

FASTQ files containing reads produced by Illumina HiSeq 2000 were examined by FastQC for quality control. All samples used in this study were Q20 > 95%. The 5′ ends of sequencing reads of samples S3–S6 (by WGA4) were trimmed 32 bp by Bowtie software (Bowtie parameter: *-5 32*), because they contained the amplification primers. Reads were aligned to mouse genome (GRCm38/mm10 version, downloaded from the UCSC Genome Browser) with Bowtie (version 2.1.0) with parameters *-I 200 -X 300*. Next the samtools mpileup function was employed to prepare consensus genotype files (subcommand: *mpileup2cns*) for variant detection. Variants were called by VarScan with default parameters^13^. In the default settings of VarScan, at least eight reads are needed to cover a base to call a variant, and the *P*-value threshold of calling a variant was 0.01. Variants with allele frequency less than 75% were called heterozygous. Otherwise they were assigned to homozygous variants.

### Analysis of RNA sequence data

RNA sequence data were aligned to mouse genome (GRCm38/mm10 version) using Tophat (version 2.0.10) with default parameters^14,15^. Then the samtools mpileup function was applied to mRNA-seq sam files to prepare consensus genotype files (subcommand: mpileup2cns) for variant detection. Variants in transcriptomes were called by VarScan^13^. Using the default setting of VarScan, at least eight reads should cover a base to call a variant, and the *P*-value threshold of calling a variant was 0.01. Gene expression level was calculated as an FPKM value by Cufflinks, with an Ensembl gene annotation gtf file^16^. The file (GRCm38/mm10) was downloaded from the Ensembl Genome Browser and only protein-coding and lncRNA (long non-coding) genes were selected^17^. The most highly expressed genes were functionally annotated by DAVID^18^. We submitted a list of Ensembl IDs of these genes to DAVID and checked “GOTERM_BP_ALL”, “GOTERM_CC_ALL”, “GOTERM_MF_ALL” and “KEGG_PATHWAY” to obtain the enriched items for the most highly expressed genes. The results are shown in Supplementary Tables 4–6.

### Integrated analysis of RNA editing sites

First we searched DNA-RNA mismatches from genomic positions with both genome and transcriptome covered by at least eight uniquely mapped reads. The variants failed by strand-filter of VarScan were discarded. Meanwhile we excluded heterozygous loci (for both exome-seq and mRNA-seq results) to eliminate potential sequencing bias and allele specific expression. We also discarded the RES candidates with more than one mismatch type (for example, DNA is “A” in S1 and S2, but RNA is “C” in S1 and “G” in S2). Next the RES candidates were compared with liver exome-seq results, and the ones found to be heterozygous in liver were discarded. Because MII oocyte is haploid while liver cell is diploid and it is possible an RNA transcript in MII oocyte was transcribed earlier in primary oocyte from the other homologous chromosome, but separated from that chromosome after meiosis I.

## Results

### Exome coverage in single isolated oocyte nuclei, single polar bodies and bulk oocytes is similar

**Figure 1:**
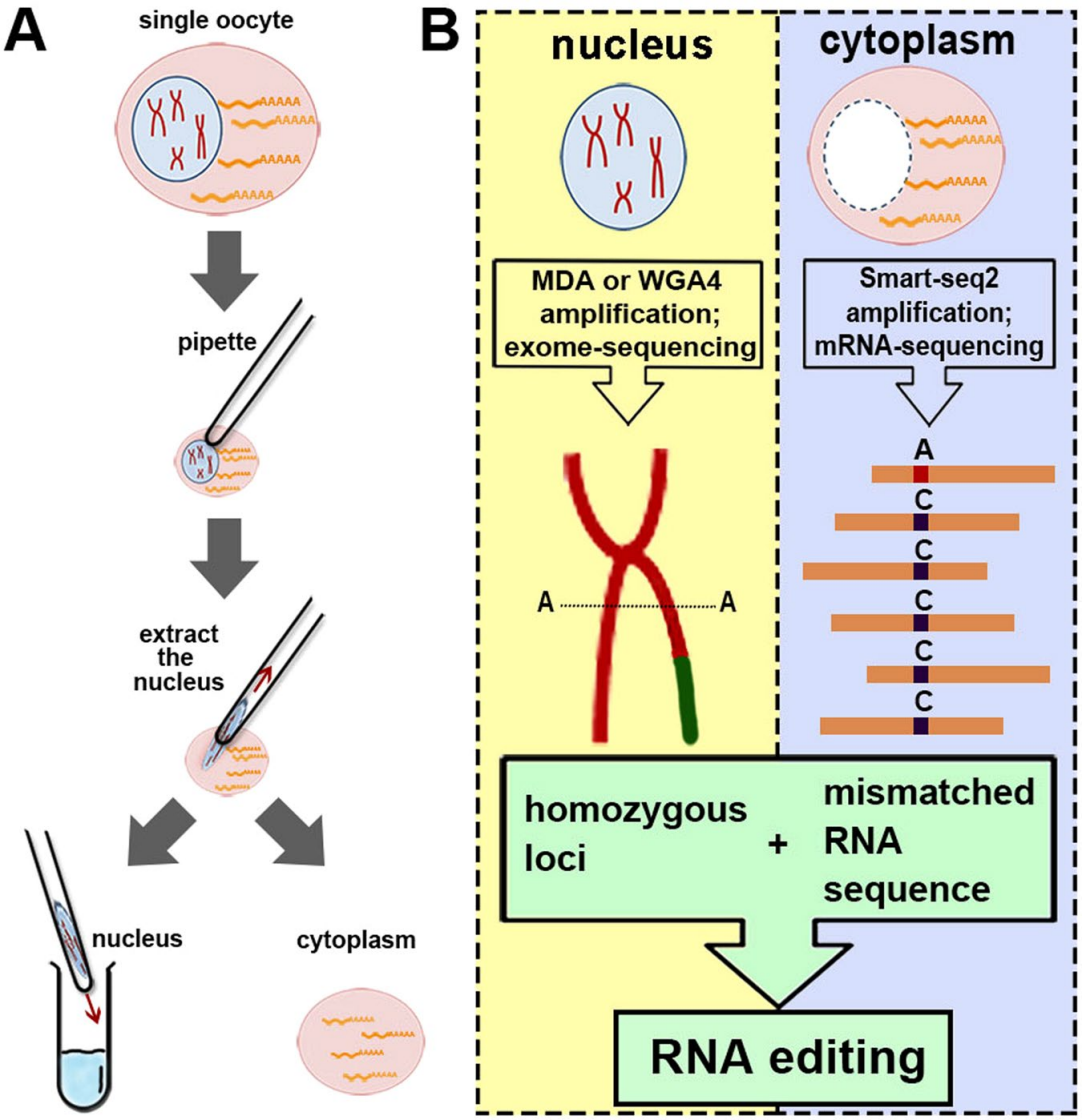
Experimental and analytical workflow. (**A**) Micromanipulation was used to physically separate nuclei from cytoplasm of single cells. (**B**) With exome-sequencing data for single nuclei and mRNA-sequencing data for the cytoplasm of the same cells, RNA editing could be detected at homozygous sites.

In brief, we extracted the nuclei from six mouse secondary oocytes (Sample ID: S1-S6) and obtained the PB1 counterparts (Sample ID: P1-P6; Fig. 1A). Because exome-seq result can well correlate with the transcriptome data, with much lower sequencing cost compared to whole genome sequencing, we performed exome sequencing on six individual oocyte nuclei and their counterpart PB1s (S1 to S6 and P1 to P6, Fig. 1A). Then single-cell mRNA-seq was performed on six enucleated cells (S1 to S6) (Fig. 1B; A summary of sequencing data is available in Supplementary Table 1). Exome coverage for isolated oocyte nuclei is more than 90% in S1 and S2 (amplified by MDA amplification method). Exome coverage of isolated nuclei and single whole cells (SW1-SW3) are similar (Supplementary Table 1), suggesting that the isolated nuclei can well represent the single whole oocyte with minimal loss of DNA.

### Heterozygous variants are observed in six individual haploid MII oocytes

**Figure 2:**
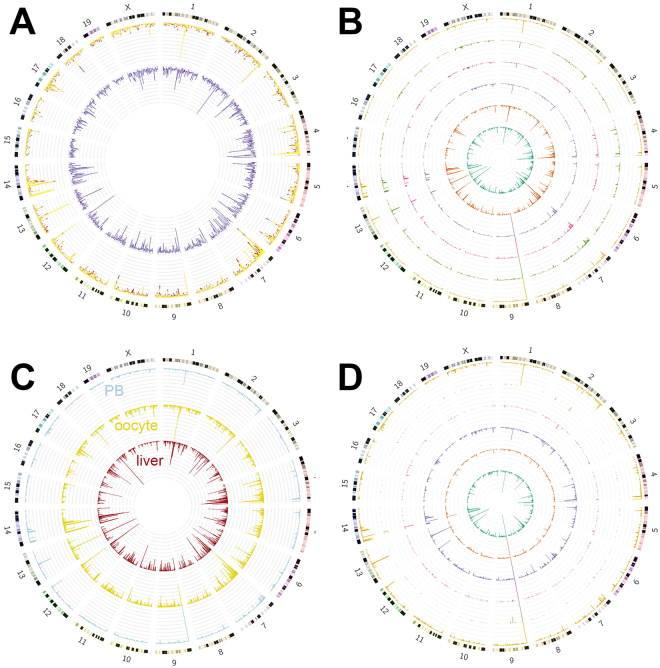
Distribution of variants on the mouse genome. (**A**) The outer bars show the number of heterozygous variants in each 1 Mb genomic region, with yellow for recombined heterozygous variants and red for non-recombined heterozygous variants. The inner purple bars show the ratio of heterozygous sites to homozygous sites in each 1 Mb genomic region. (**B**) Each circle shows the number of recombined heterozygous variants in 1 Mb genomic regions, detected in a single nucleus. (**C**) Numbers of heterozygous sites in each 1 Mb genomic region in liver tissue, oocytes and PB1 (first polar body) counterparts are shown. (**D**) Each circle shows the number of recombined heterozygous variants in 1 Mb genomic regions, detected in a single PB1.

Single-nucleus sequencing showed that six oocytes were genetically different due to meiosis and meiotic recombination. In the pooled exome-seq data of S1 to S6, VarScan detected 726,525 variants from the 0.93 Gb (34.2%) of the mouse genome covered^13^. Heterozygosity was detected in 436,535 variants and for 290,264 (66.5%) of these heterozygous variants both alleles were present in the genome of a single oocyte (36,000 to 98,000 heterozygous loci in S1 to S6). Although these oocytes are haploid, the heterozygous loci could be explained by meiotic recombination. The oocytes are at meiosis II and each chromosome has two sister chromatids; therefore, genetic recombination between homologous chromosomes during meiosis I leads to heterozygous loci in a haploid oocyte^19^. Majority of heterozygous variants were exchanged to the homologous chromosome, at least in one cell, based on the number of heterozygous loci in single oocytes (yellow for heterozygous variants; red for homozygous variants in Fig. 2A). This indicated that oocytes underwent extensive recombination during meiosis II. For each oocyte, the distribution of heterozygous loci along the mouse genome was generally similar, with a few exceptions (Fig. 2B). In Fig. 2B, each circle is variant distribution of a single cell. The inner two cells (S1 and S2), which were amplified by MDA, contained more reads and a higher coverage rate compared with other four cells amplified by DOP-PCR.

Moreover, the sequencing result of the bulk liver cells (Sample ID: BL) of the same mouse was served as the reference genome. We also sequenced the genome of polar body 1 counterparts (P1-P6) for oocytes to confirm that the heterozygous variants detected from single haploid oocytes are due to meiotic recombination and to accurately pinpoint the exchanged regions during recombination. All experiments in this study are summarized in Supplementary Table 2.

As seen from Fig. 2C, the distribution of heterozygous loci in oocytes, PB1 counterparts and liver cells is quite similar. Figure 2D shows the distribution of recombined heterozygous loci in each PB1, which match the pattern of that in oocytes, shown in Fig. 2B. This highly consistent result for heterozygous loci and recombined heterozygous loci showed that the exome information was accurate. On the other hand, only <0.05% (~46,000) of homozygous loci in oocytes were designated as heterozygous loci in liver by exome-seq data and <0.31% of heterozygous loci in oocytes were found as homozygous loci in liver cells, suggesting that the exome-seq data in oocytes faithfully reproduced the exome information, without biased allele selection or low sequencing quality.

In general, the exome-seq experiments conducted on the secondary oocytes, the first polar bodies and bulk liver cells of the same mouse provided a reliable genome reference of the individual mouse, permitting the following integrative analyses.

### A similar number of expressed genes were detected in single enucleated oocytes, single whole oocytes and bulk oocytes

**Figure 3:**
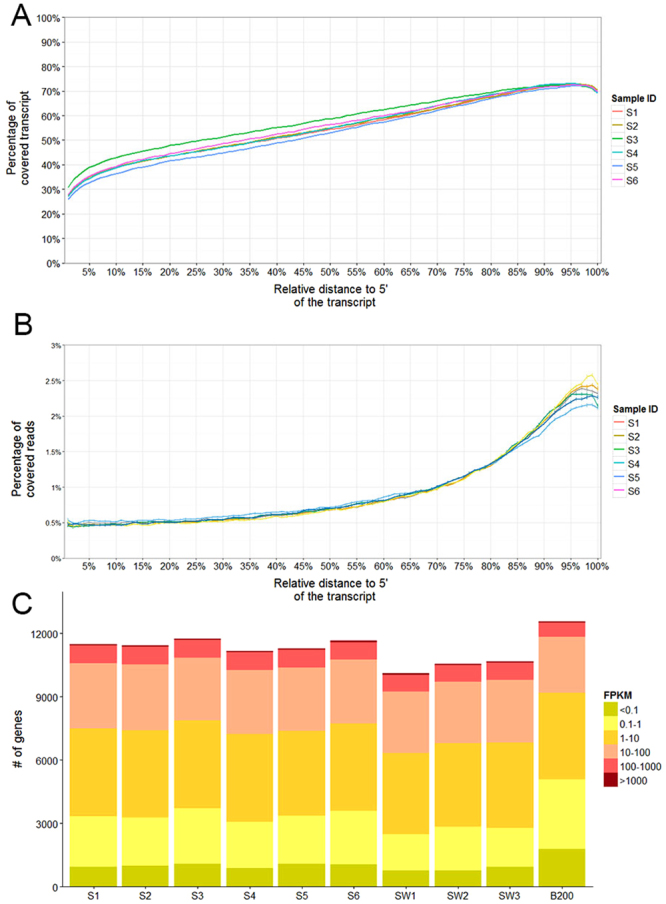
Coverage of mRNA-seq reads across transcripts. (**A**) The percentage of genomic region covered by mRNA-seq reads in each 1% of a transcript (compared to 0.01 × (length of this transcript)). (**B**) The percentage of mRNA-seq reads in each 1% of a transcript (compared to all reads in this transcript). (**C**) Distribution of fragments per kilobase of transcript per million mapped reads (FPKM) values.

We compared the number/abundance of transcripts and calculated the correlations of gene expression values in single enucleated oocytes (S1–S6), single whole oocytes (SW1–SW3, from the same mouse as S1–S6) and 200 oocytes (B200; from multiple mice) (summary of all experiments is shown in Supplementary Tables 1 and 2). Reads produced by mRNA-seq in S1 to S6 were distributed uniformly across all transcripts (Fig. 3A), indicating that the amplification method, Smart-seq2, could recover full-length mRNA transcripts^20^. Measuring the proportion of transcripts covered by sequencing reads showed that the coverage rate of the 3′ regions of transcripts was high, but overall the coverage rate was between 25% and 75% for the entire transcript, suggesting that the 5′ regions of transcripts were also recovered during cDNA amplification (Fig. 3A). When calculating the frequency of reads located in each 1% of the transcript, we found fewer reads, but not none, were located at the 5′ end of transcripts (Fig. 3B). We further examined the coverage rate and read frequency along mRNA transcripts by grouping transcripts by length, and the results clearly demonstrated that for longer transcripts the 3′ bias was greater (Supplementary Fig. 1)^21^.

In each single oocyte (S1–S6 and SW1-SW3), more than 10,000 protein-coding and lncRNA genes were detected, and with a more stringent standard, fragments per kilobase of transcript per million mapped reads (FPKM) greater than 0.1, we still identified approximately 10,000 genes expressed in each single oocyte (Fig. 3C). The number of expressed genes in single oocytes was fewer than in bulk oocytes, as expected, and the genes expressed in B200 but not in single oocytes generally showed small FPKM values (Fig. 3C). Dynamic transcription regulation and transcription bursts contribute to transcriptome heterogeneity; therefore, a pool of 200 cells is likely to have more genes expressed compared with single cells analysed at similar sequencing depth, probably with a low expression level (Supplementary Table 3)^22,23^. Surprisingly, we found a greater number of expressed genes in enucleated oocytes (S1–S6) compared with whole single oocytes (SW1-SW3), and all the genes expressed in whole oocytes were expressed in enucleated oocytes. This might be due to the slightly higher sequencing depth for enucleated oocytes (Supplementary Table 3), averaging 25 million (1.5%) more mapped bases compared with whole oocytes. It was found that, with a similar number of reads for each sample (less than 40 million), a greater number of expressed genes may be discovered given a higher number of mapped bases^24,25^. In addition, cell-to-cell differences and a changing environment may cause variation in the number of expressed transcripts^26^. By defining “expressed genes” as having FPKM values of at least 1, we identified 526 genes expressed in whole oocytes but not in enucleated oocytes. In addition, the difference in expression level of these genes between the two cell states was quite small, with a median of 0.97 FPKM (Supplementary Fig. 2). This suggests that extracting the nuclei resulted in a trivial loss of poly(A) RNA transcripts.

For the mapped reads from S1–S6, 17.0–19.5% were intergenic and 5.5–6.0% were located in introns. Similarly, 17.8–19.9% of the mapped reads from SW1-SW3 were in intergenic regions. However, 6.7–7.3% were intronic, a slightly higher compared with that of single enucleated cells. This may result from unspliced transcripts in the nuclei. In sample B200, around 10% of mapped reads were in introns, and 34.1% were not mapped to ensemble genes, suggesting a high level of transcription dynamics.

In general, the number of expressed genes in enucleated oocytes is similar as that in the single whole oocytes, indicating that the mRNA content in cytoplasm is well preserved. Moreover, more intronic sequences are observed in single whole cell suggesting that some of the precursor genes are only retained in cell nucleus, and are lost in the enucleated cells.

### Gene expression levels in single oocytes are highly correlated

DAVID analysis showed that the 100 most highly expressed genes in S1 to S6 were enriched for some GO terms, including cell cycle (GO:0007049), cell division (GO:0051301) and gamete generation (GO:0007276), and for the biological pathways of oocyte meiosis and cell cycle (Supplementary Table 4)^18^. The 100 most highly expressed genes in SW1 to SW3, and B200 were also enriched for similar terms (Supplementary Tables 5 and 6), indicating that the oocytes in these samples functioned normally and that the transcripts of function-related genes were abundantly expressed.

**Figure 4:**
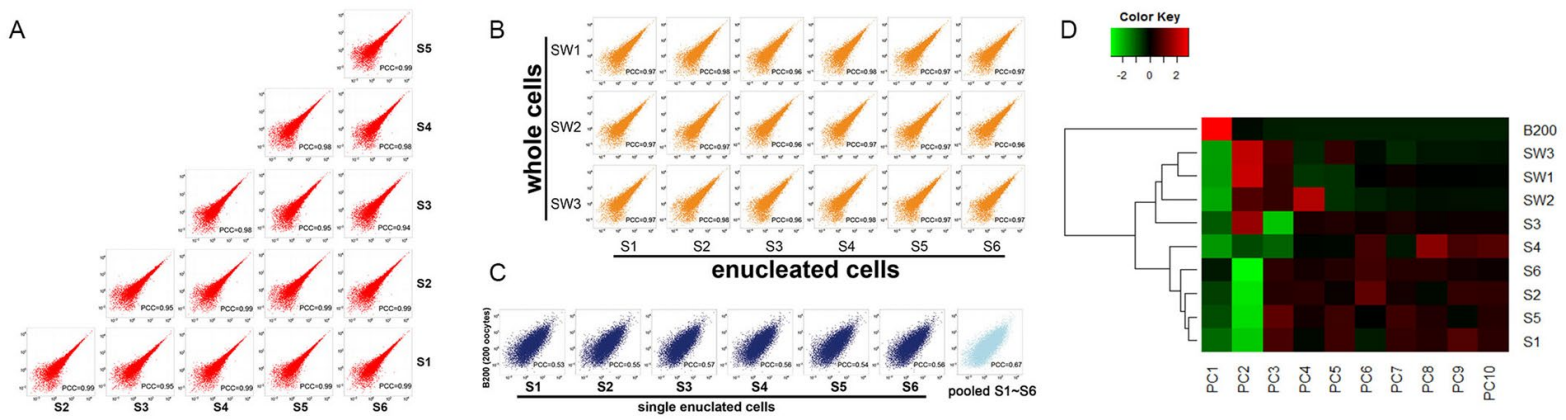
Correlation of expression values between samples. (**A**) Scatterplot of expression values of single enucleated oocytes. (**B**) Scatterplot of expression values of single enucleated oocytes and single whole oocytes. (**C**) Scatterplot of expression values of single enucleated oocytes and bulk oocytes. (**D**) Principal component analysis was performed on expression values of single enucleated cells, single whole cells and bulk cells. The heatmap is drawn based on resulting principal components.

Cell-to-cell variability in transcriptomes was measured in previous studies by calculating the correlation coefficient of expression values between single cells^23,27^. Here we used the same method and confirmed that single oocytes from the same organism displayed almost identical gene expression profiles. The Pearson’s correlation coefficient (PCC) values between any pair of enucleated oocytes was greater than 0.94 (*P*-value < 2.2E-16, Pearson’s correlation test, Fig. 4A). In addition, the PCCs between FPKM values of enucleated oocytes (S1–S3) and whole oocytes (SW1-SW3) were also great (*P*-value < 2.2E-16, Pearson’s correlation test, Fig. 4B). In addition to similar number of detected genes described above, this high correlation further shows that profiling transcriptomes in enucleated oocytes faithfully recapitulates the findings in whole oocytes. In contrast, the correlations between enucleated oocytes and bulk oocytes were much lower (PCCs were smaller than 0.6; *P*-value < 2.2E-16; Fig. 4C). This great difference in PCC values suggests that cell-to-cell variability is much smaller than the heterogeneity among different individuals (Fig. 4D). In short, these results indicated that this method is highly reproducible, as can be observed from the highly correlated gene expression in six enucleated single cells.

### Various types of RNA editing were detected by transcriptome-exome sequencing of individual cell

**Figure 5:**
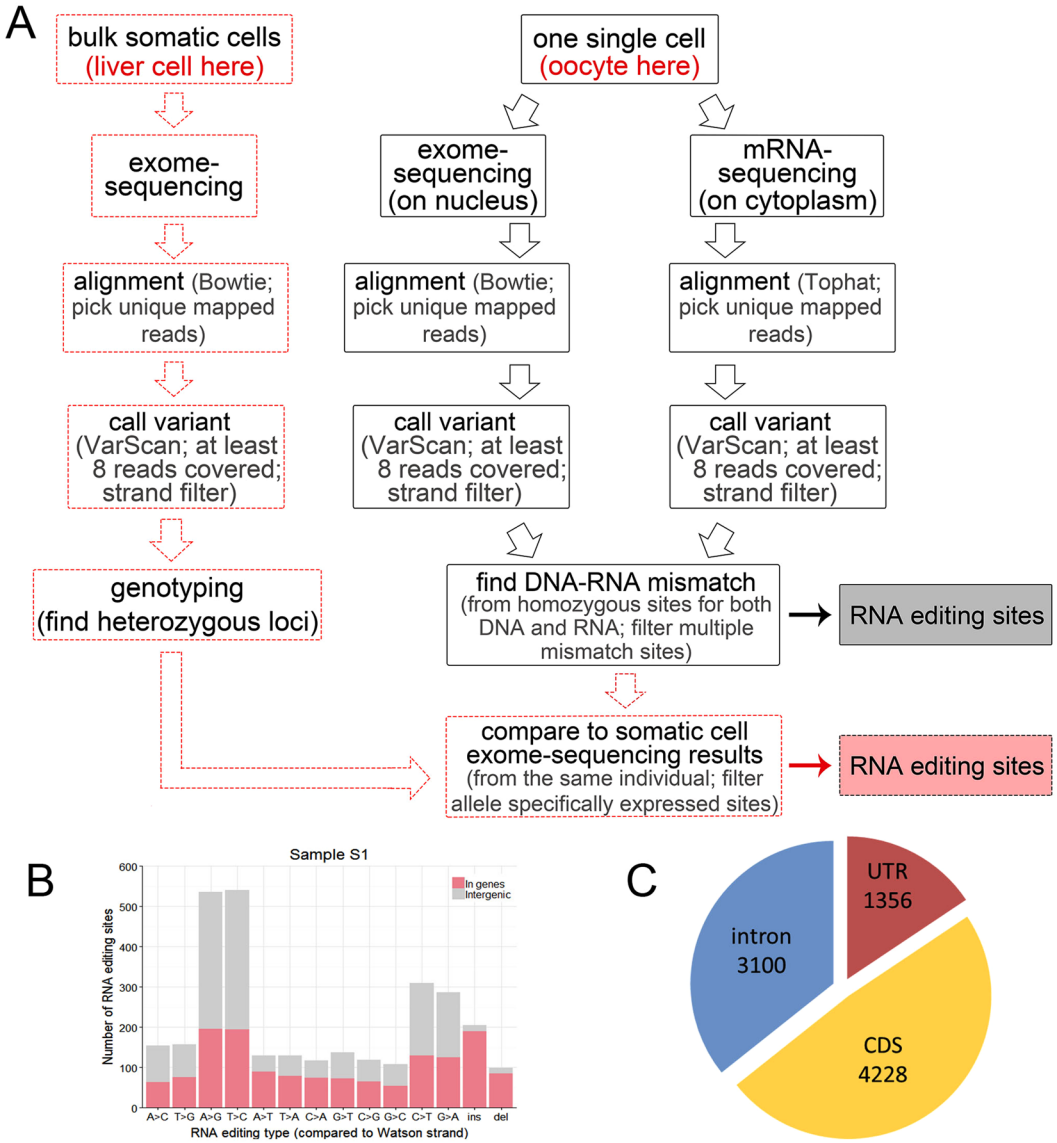
Methods and results of RNA editing site (RES) detection. (**A**) The pipeline for RNA editing site detection. (**B**) Number of RESs in Sample S1. The editing type is relative to DNA sequence on the Watson strand. We used a non-strand-specific mRNA-seq method; therefore, it is not clear whether these mRNA transcripts are from the Watson or Crick strand. Ensembl gene annotation is used as a reference. (**C**) Distribution of the RESs located in genes.

Our method is able to detect the DNA-RNA regulation in single cell at single-nucleotide resolution. We here analysed RNA editing of individual oocytes to demonstrate one application of this method. RNA editing occurs in prokaryotes, plants and animals, contributing markedly to transcript diversity and cellular function^28^. To detect or confirm RNA editing sites (RESs), the genome and transcriptome of the same organism are compared and mismatched sites selected as candidates^28–33^. Here we managed to locate RESs in single cells using the same method (Fig. 1B). A stringent standard was applied to find true RESs when comparing the exome-seq and mRNA-seq reads (Fig. 5A). We picked only uniquely mapped reads, filtered the reads showing strand specificity, and required the DNA and RNA sequences at a site to be homozygous in the cell. This is because sites showing homozygous DNA and heterozygous RNA sequences might result from biased exome-seq on two alleles, while sites showing heterozygous DNA and homozygous RNA sequences might result from monoallelic expression. Furthermore, we discarded RES candidates which showed multiple forms of mismatch^34^.

In each single cell, we detected 1,051–3,385 RESs (Supplementary Table 7) of various types (Fig. 5B; Supplementary Fig. 3). A-to-G and T-to-C RESs accounted for 26–35% of all sites in our study, which is consistent with previous findings that A-to-I (Inosine is decoded as Guanine) edits were most common^28,31,32^. Previously only a handful of other types of RNA editing were detected, but a list of non-A-to-G RESs was identified recently^35–39^. mRNA-seq experiments in this study were not strand-specific; therefore, we used Ensembl gene annotation to find the potential sense-strand of the transcripts and determine the actual editing type (if mismatch is A-to-G and gene is on Watson strand, RES is A-to-G; if mismatch is A-to-G and gene is on Crick strand, RES is T-to-C). By mapping the RESs to protein-coding and lncRNA genes, we found that nearly half of the RESs were intergenic, in agreement with previous findings^32^ (Fig. 5B; Supplementary Fig. 3) and 2,068 genes containing RESs were evenly distributed on Watson and Crick strands. Half of these RESs were in coding regions (Fig. 5C), and only 16 RESs were in start and stop codons. A-to-G but not T-to-C is predominant in RNA editing in mammals; therefore, a similar number of A-to-G and T-to-C RESs located in genes suggested that antisense transcripts were also subject to RNA editing.

RESs identified through DNA and RNA comparison include false positives caused by SNPs and somatic mutations^33^. Here we used single-cell sequencing data of both DNA and RNA to exclude false positive findings caused by SNPs and somatic mutations, which is usually a problem when bulk cell data or only RNA data is used. However, because oocytes are haploid cells derived from diploid cells, the RNA produced in diploid might remain in oocytes. Compared with the exome sequences of liver cells, only 3.8% to 6.4% of the RESs were found to be heterozygous. These RNA sequences are possibly synthesized from the one chromosome in diploid cells, and remain in the cytoplasm with the nucleus containing the homologous chromosome.

**Figure 6:**
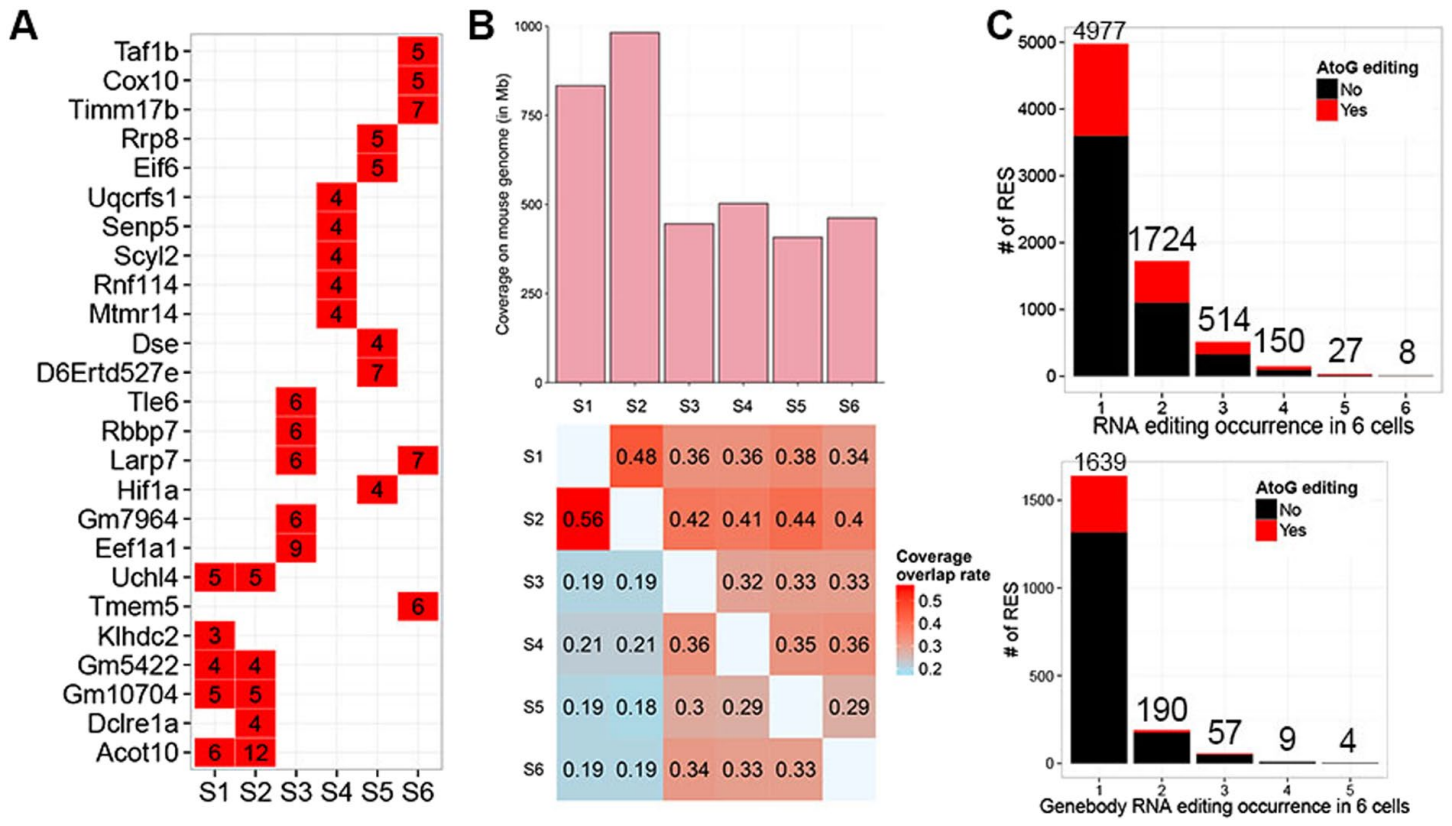
Summary of RES number in single cells. (**A**) Top genes with most RESs. Numbers in red square are detected RES number in a gene from a sample (single cell). (**B**) Genomic coverage size (upper) and rate of genomic coverage overlap between two samples (lower). Lower heatmap shows the rate of coverage overlap of two samples over coverage of column sample. (**C**) Occurrence of RESs detected in six single cells. Upper plot is for all RESs, and lower plot is for RESs in genes.

From the most edited genes in each cell, we observed that S1 and S2 showed a similar pattern, while other cells displayed a distinct RNA editing pattern (Fig. 6A). Because S1 and S2 were amplified using MDA method while the other four cells were amplified by WGA4 kit, the amplified region in two groups were different, which lead to a different pattern of RESs distribution in different cells. Although the genome coverage is similar among six samples, the common genome coverage is low (Fig. 6B). At most two samples have half of the covered region in common (S1 and S2). It’s not common to get the sequence of a genomic position in all samples by exome-seq when a single copy of genome is used as the template, so detecting RNA editing in all six samples is difficult. However, we do observed some RESs in more than one cell, and even occurring in all six cells (Fig. 6C and Supplementary Table 8).

## Discussion

Single-cell genome analysis, single-cell transcriptome analysis, and integrated genome-transcriptome analysis of both single cell and bulk cells have been conducted. Here we developed an integrated analysis of single-cell exome and transcriptome in large-sized cells to discover the connection between genome and transcriptome of the same cell. To obtain gDNA and RNA, we first manually extracted nucleus by microinjection system from single oocyte for exome-seq, leaving the enucleated oocyte for mRNA-seq. This method could effectively and efficiently separate gDNA and RNA, without the need to isolate RNA from a mixture of these two molecules by electric fields or magnetic beads^1,3^. Then we used well-developed amplification methods (WGA4 and MDA for gDNA; Smart-seq2 for RNA) to generate sequencing libraries based on the small quantities of starting gDNA or RNA. We obtained wide coverage (up to 34.2% of the mouse genome) and high depth (more than 720,000 variants were detected) from exome-seq, and abundant poly(A)-tailed transcripts from mRNA-seq (more than 10,000 genes), as compared with current methods^20,21,40–43^. The micromanipulation technique here used to separate gDNA and RNA is quite straightforward and easily applied, which is much less demanding compared with microfluidics and isotachophoresis (ITP)^2,3^. This method is selectively appropriate for large-sized cells, including oocytes, large neurons, tumour cells *etc*.

Recently, two innovative methods named DR-Seq and G&T Seq were developed to sequence gDNA and mRNA of the same single cell^4,5^. Different from our method, DR-seq begins with pre-amplification of single-cell DNA and mRNA within a single tube without physical separation of nucleus and cytoplasm. We do agree that this method is particularly useful when a large number of single cells are used for primary analysis. However, as DR-seq does not pre-separate gDNA and mRNA, it’s impossible to distinguish the source of exome sequences or study both the CNV and SNV in the exome sequences, not to mention the DNA-RNA correlation in single cell. Besides, DR-seq requires several steps of amplifications using different primers or indexes. Therefore, the quantification of raw DNA and RNA molecules requires accurate and efficient primer ligation and removal, otherwise the reads may be falsely assigned. G&T-seq adopts a different approach to separate genomic DNA and mRNA. Cell is lysed thoroughly, and then mRNAs are enriched and separated from genomic DNA using biotin-labelled oligo (dT) followed by precipitation using streptavidin beads. This method is also appropriate for large-scale screening of multiple single cells. However, G&T-seq still suffers from high variability and relatively low efficiency of capturing a tiny amount of raw RNA from single cell. Firstly, the required reaction volume is relatively high, because it needs to be mixed with microbeads. The reaction volume can be as high as 20–50 μl before any amplification, but large volume will lead to low efficiency of capturing a tiny amount of gDNA and RNA from single cell. Moreover, since genomic DNA are attached to the beads, several times of washing could lead to loss of the raw material. Therefore, the number of expressed genes detected by this method is highly variable, ranging from 4,000 to 11,000^5^. A large amount of genomic DNA and mRNA could be lost during beads separation and elution steps.

We performed mRNA-seq in single whole oocytes (SW1-SW3) and enucleated single oocytes (S1–S6). We compared transcript abundance and found trivial differences and high correlation of the two groups. These results demonstrated that the transcriptome in an enucleated cell was comparable to the transcriptome profile in a whole cell. We believe that this integrative analysis of the genome and transcriptome from a single cell will benefit studies addressing genotype-phenotype relationships.

For demonstration of the potential application of our method, we integrated gDNA information with mRNA transcript abundance of individual cell to accurately measure allele expression frequency and locate RESs. For allelic specific expression studies based on RNA profiling of bulk cells, complicated computational models or well-designed animal lines with known genotypes are required^44–46^. Transcriptome analysis of single cells of known genotype, enabled insightful findings concerning random and dynamic monoallelic expression to be deduced^47^. Here we simultaneously sequenced the gDNA and RNA of six single oocytes individually. Previous studies investigating RESs have mostly focused on A-to-I RNA edits, but here the information of both gDNA and RNA from same single cells enabled us to find many RESs of other types (Fig. 5)^28–31^. However, the resulted RESs were not always detected in all samples, which might be caused by low genomic coverage overlap among samples due to low specificity of probe capture in exome-seq, or by the intrinsic nature of rare occurrence of RNA editing^48^. Another interesting topic in integrative genomic studies is regulatory variants, the study of which benefits from simultaneous DNA- and RNA-sequencing in a single cell^49^. Here, we tried to find regulatory variants in single cells by locating variants in the gene bodies and promoter regions of highly expressed genes, but no significant regulatory variants were found in the six cells analysed. This integrated analysis will be applicable to other cell types, such as normal and tumour cells from the same organism^50^.

As mentioned above, our method is limited to cells of a large size. Here we used mouse oocytes, which are relatively large for eukaryotic animal cells with a diameter of 50–70 microns^51–53^ (most animal cells are 10–30 microns). Theoretically, micromanipulation can be applied to any mammalian cell, but smaller cells present more difficulties^54^. With the microcapillary needle between 0.5 and 5 microns and the holding needle between 10–50 microns in diameter, selecting large cells, such as oocytes, neurons and tumour cells, is recommended^55–60^. During nucleus extraction there is a loss of cytoplasm, which adheres to the tip. Another limitation is that the transcripts in the nucleus were not sequenced, which results in the loss of the information of mRNA precursors.

In contrast, although our method is not suitable for large-scale parallel study, it is quite good to study some specific cells in depth, particular large-sized cells. Our method is more like a complementary step following the original screening steps by either the DR-seq or G&T-seq. The advantages of current approach includes: (1) DNA and mRNA are totally separated and kept intact before amplification or other procedures, which avoids contamination and reduces the nucleic acid degradation. (2) It is free to choose any amplification method for the isolated nucleus and enucleated cell according to the aim of a study, such as single-cell methylation sequencing. (3) The performance of our method is more consistent, we have more starting material, and the amplification efficiency is higher. For example, we are able to recover more than 90% of exome sequence for a single isolated nucleus. These advantages allow us to analyse single-cell genome, transcriptome and possibly epigenome in an integrated way. It will facilitate a more complete understanding of the extent, function and evolution of cellular heterogeneity in normal development and disease processes.

## Conclusions

In summary, we simultaneously sequenced the exome and transcriptome of large-sized single cells. The exome-seq data and mRNA data, showing good reproducibility and high coverage, suggested that this integrated DNA-RNA analysis method can well preserve DNA in the isolated nucleus and mRNA in the cytoplasm. Using strict selection criteria, we detected hundreds of RNA editing sites in individual single oocytes with this unprecedented method of separating DNA and RNA in one cell. Our study will improve the understanding of DNA-RNA regulation mechanism by directly correlating the genome sequences and mRNA sequences of a single cell.

## Declarations

### Availability of data and material

Exome data and RNA-seq data are available from GEO database under accession number [GEO: GSE94813].

## Electronic supplementary material

Supplementary material

Supplementary Table 4

Supplementary Table 5

Supplementary Table 6

Supplementary Table 7

Supplementary Table 8
