# Supplementary material for: Integrated sequencing of exome and mRNA of large-sized single cells
Source: Sci Rep. 2018 Jan 10;8:384. doi: 10.1038/s41598-017-18730-y (PMC5762704; doi:10.1038/s41598-017-18730-y)
Supplement: Supplementary file 1 — Supplementary material [file 41598_2017_18730_MOESM1_ESM.pdf]

Supplementary Tables and Figures

Integrated sequencing of exome and mRNA of large-sized single cells

Lily Yan Wang[#], Jiajie Guo[#], Meng Zhang, Wei Cao, Jiankui He*, Zhoufang Li*

Department of Biology, Southern University of Science and Technology, 518055, China

[#]These authors contributed equally.

*Corresponding authors: lizf@sustc.edu.cn; hejk@sustc.edu.cn

Supplementary Table 1. Summary of single-cell sequencing data.

	S1	S2	S3	S4	S5	S6
	exome-sequencing (90bp paired-end)					
**Clean reads (in million pairs)**	118.9	147.5	86.0	92.2	88.0	91.8
**Clean bases (in Gb)**	21.4	26.6	10.0	10.7	10.2	10.6
**Mapped reads (in million)**	231.6	287.2	146.7	156.4	148.8	153.3
**Mapped bases (in Gb)**	20.8	25.8	8.5	9.1	8.6	8.9
**Mapping rate**	97.4%	97.3%	85.3%	84.9%	84.6%	83.5%
**Genome coverage (in Gb)**	1.39	1.45	0.69	0.75	0.71	0.64
**Genome coverage rate[$]**	50.9%	53.4%	25.3%	27.6%	25.9%	23.6%
	mRNA-sequencing (49bp single-end)					
**Clean reads (in million)**	36.4	36.5	37.4	36.9	35.3	37.1
**Clean bases (in Gb)**	1.8	1.8	1.8	1.8	1.7	1.8
**Mapped reads (in million)**	34.4	34.4	35.5	34.8	33.0	34.8

**Mapped bases (in Gb)**	1.7	1.7	1.7	1.7	1.6	1.7
**Mapping rate**	94.4%	94.3%	94.9%	94.4%	93.5%	93.8%
**Unique mapped reads / mapped reads**	87.1%	87.4%	87.8%	86.6%	86.8%	86.9%
**# of genes with FPKM greater than 0***	11479	11424	11750	11166	11271	11638
**# of genes with FPKM greater than 1***	8143	8144	8039	8090	7915	8047

[$] *Mouse genome size is 2,725,537,669bp (mm10).*

** Gene coordinates are from Ensembl annotation on protein coding genes and lincRNA genes.*

There are 23,975 protein coding and lincRNA genes from Ensembl annotation.

Supplementary Table 2. Summary of all experiments in this study.

Experiment	# of cells in each sample	Cell type	Sample IDs	Purpose
exome-seq	1	Nuclei of 6 oocytes	S1 – S6	Answer the main question
mRNA-seq	1	6 enucleated oocytes	S1 – S6	Answer the main question
exome-seq	bulk	Liver cells	BL	Compare with oocyte genome
exome-seq	1	6 PB1s (counterparts of above oocytes)	P1 – P6	Compare with counterpart oocyte
mRNA-seq	1	3 oocytes	SW1 – SW3	Prove the small number of lost mRNAs in nucleus
mRNA-seq	bulk	Around 200 oocytes	B200	Compare with single-cell transcriptomes

Supplementary Table 3. Summary of parallel mRNA-sequencing data.

	SW1	SW2	SW3	B200
Clean reads (in million)	35.8	37.0	36.0	37.3
Clean bases (in Gb)	1.8	1.8	1.8	1.8
Mapped reads (in million)	33.6	34.6	33.7	34.0
Mapped bases (in Gb)	1.6	1.7	1.7	1.7
Mapping rate	93.8%	93.6%	93.7%	91.2%
Unique mapped reads / mapped reads	86.0%	86.9%	85.9%	81.7%
# of genes with FPKM greater than 0*	10133	10600	10722	12258
# of genes with FPKM greater than 1*	7621	7713	7897	7491

** Gene coordinates are from Ensembl annotation on protein coding genes and lincRNA genes. There are 23,975 protein coding and lincRNA genes from Ensembl annotation.*

Supplementary Table 4. Functional enrichment result by DAVID on top 100 genes in six single enucleated oocytes.

Supplementary Table 5. Functional enrichment result by DAVID on top 100 genes in three single whole oocytes.

Supplementary Table 6. Functional enrichment result by DAVID on top 100 genes in bulk oocytes.

Supplementary Table 7. List of RNA editing sites located in genes.

Supplementary Table 8. Occurrence of RNA editing sites in six oocytes.

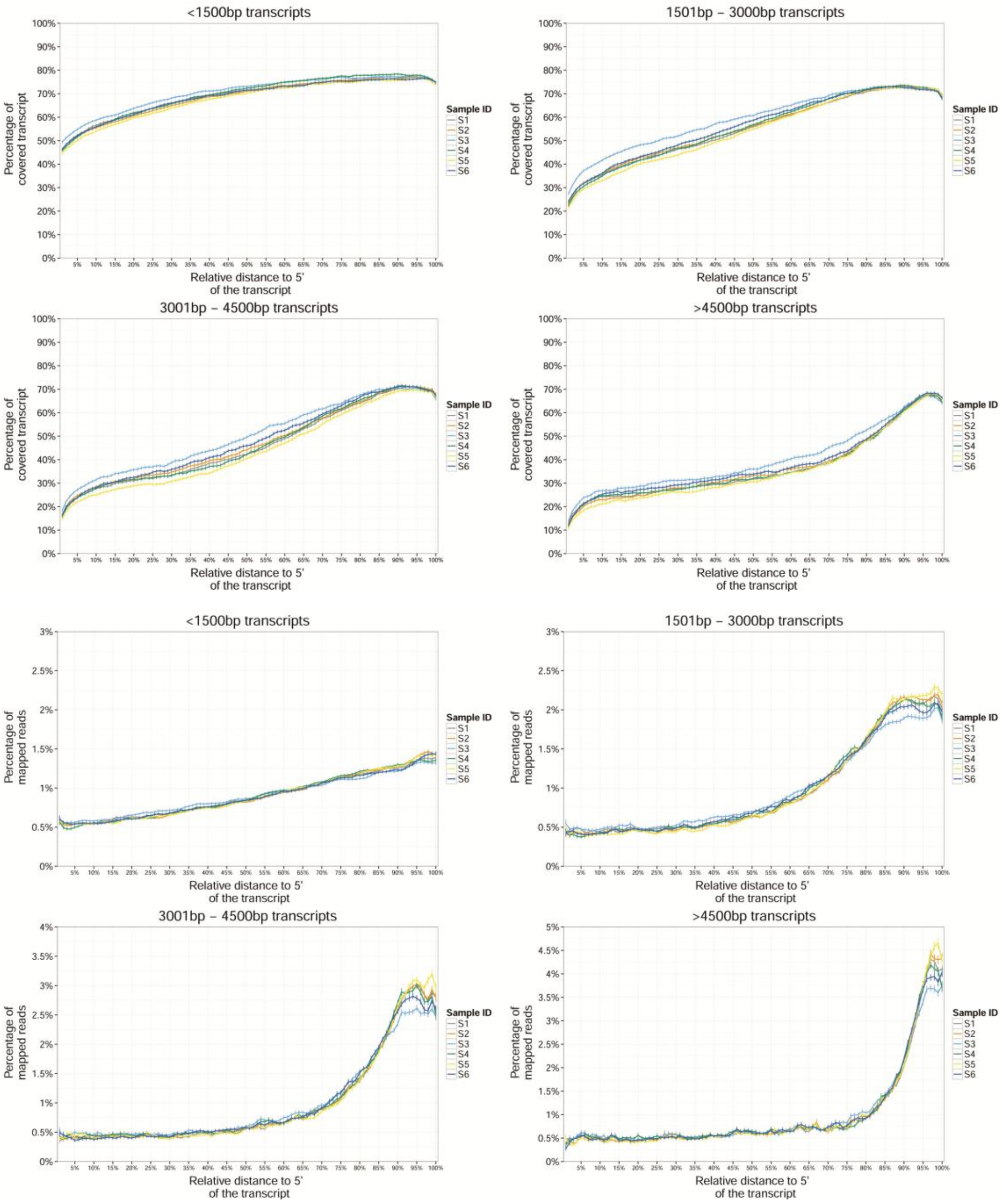

Supplementary Figure 1. The percentage of region covered by mRNA-seq reads in each 1% of a transcript and the percentage of reads number from mRNA-seq in each 1% of a transcript, for transcripts of different length. With the transcript length increasing, 3’ bias is more obvious.

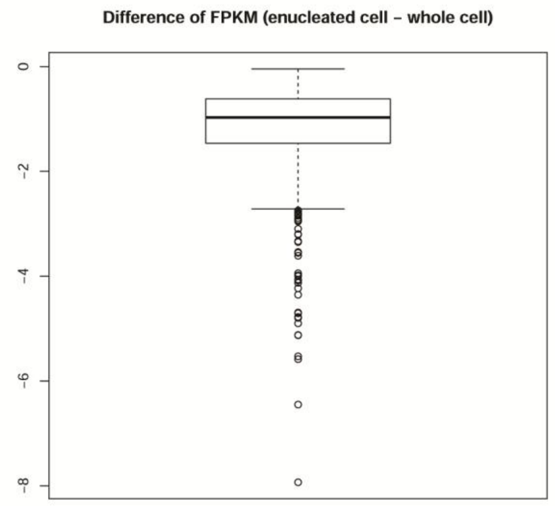

Supplementary Figure 2. A boxplot showing the difference of FPKM (whole cell minus enucleated cell) of 526 genes. These genes have FPKM values greater than 1 in whole cells and FPKM values less than 1 in enucleated cells. The median of difference is only 0.97, showing that only a small number of transcripts are lost when nuclei are removed.

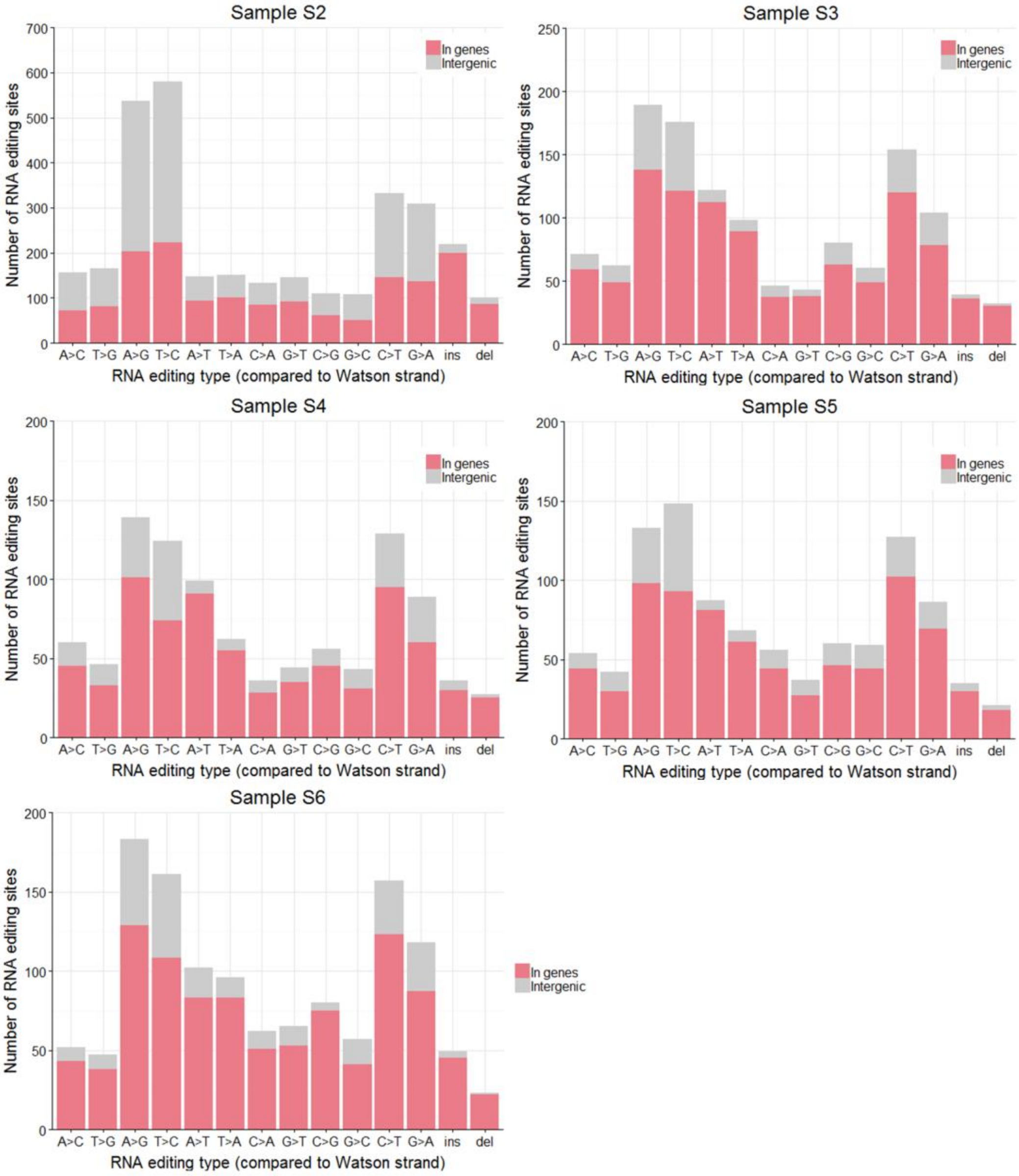

Supplementary Figure 3. Bar plots showing RNA editing types in sample S2-S6. Similar to Fig. 5b, the most frequent RNA editing type is A-to-G/T-to-C. Due to different sequencing depth in S1 to S6, the number of RNA editing sites is different.
